# MR imaging of human atherosclerosis using immunomicelles molecularly targeted to macrophages

**DOI:** 10.1186/1532-429X-11-S1-P83

**Published:** 2009-01-28

**Authors:** Vardan Amirbekian, Michael J Lipinski, Juan C Frias, Smbat Amirbekian, Karen C Briley-Saebo, Venkatesh Mani, Daniel Samber, Antonio Abbate, Juan GS Aguinaldo, David Masey, Valentin Fuster, George Vetrovec, Zahi A Fayad

**Affiliations:** 1 Harvard Medical School – Mount Sinai School of Medicine, Imaging Science Laboratories, Brigham and Women's Hospital, New York, NY USA; 2 Mount Sinai School of Medicine, Translational and Molecular Imaging Institute., New York, NY USA; 3 Emory University School of Medicine., Atlanta, GA USA

**Keywords:** Inductively Couple Plasma Mass Spectroscopy, Lipid Core, Severe Atherosclerosis, Confocal Fluorescent Microscopy, Clinical Scanner

## Introduction

Early assessment of atherosclerosis (leading cause of death in West and soon world) remains an elusive clinical goal, which if realized could lead to significant improvements in mortality and morbidity.

## Purpose

Gadolinium (Gd)-containing immunomicelles targeting macrophages improved magnetic resonance (MR) detection of murine atherosclerosis. We sought to determine if immunomicelles targeting the macrophage scavenger receptor-B (CD36) improved ex-vivo MR detection and characterization of human aortic atherosclerosis.

## Methods

Gd-containing micelles, anti-CD36 immunomicelles and Fc-micelles were created. Macrophages were incubated with fluorescent micelles and immunomielles to determine uptake via confocal microscopy and inductively coupled plasma mass spectroscopy (ICP-MS) was performed to quantify Gd uptake. Human aortic specimens with moderate to severe atherosclerosis were harvested at autopsy. Using a 1.5 T Siemens clinical scanner, T1, T2, and PDW 3-dimensional scans were performed and post-contrast scans were repeated after 24 h incubation. T1 analysis and cluster analysis were performed comparing immunohistopathology with MR images. P-values < 0.05 were considered significant.

## Results

**Figure 1:**
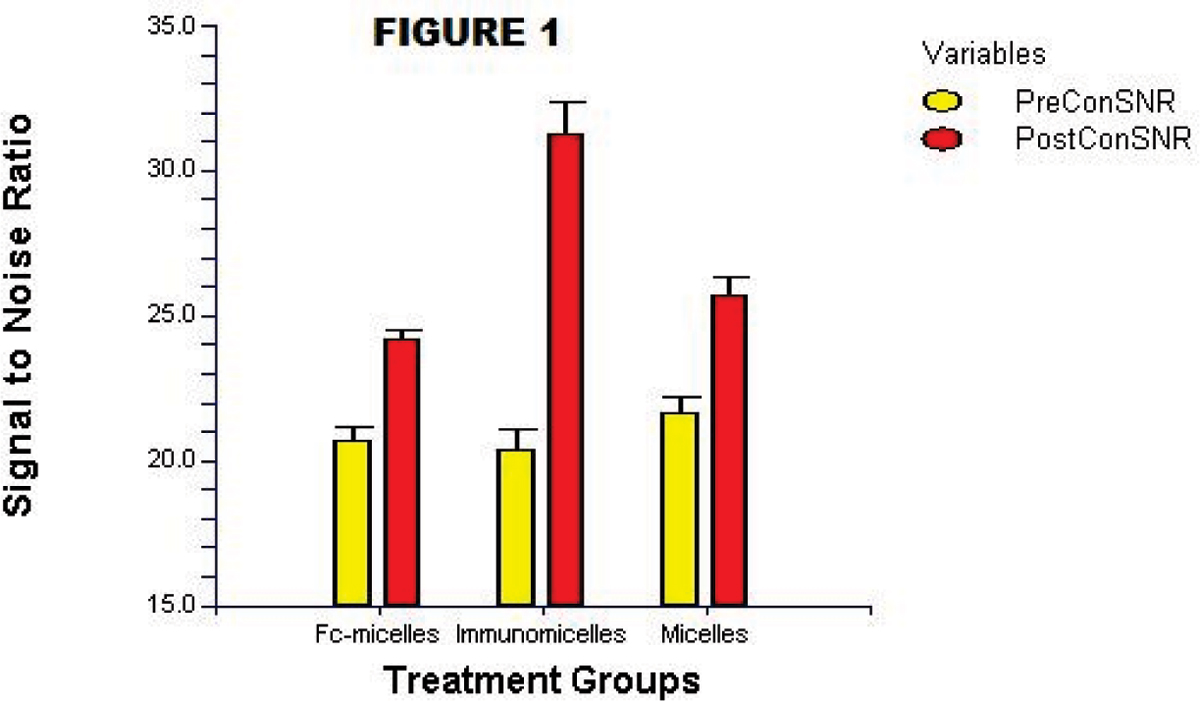
Figure 1

**Figure 2:**
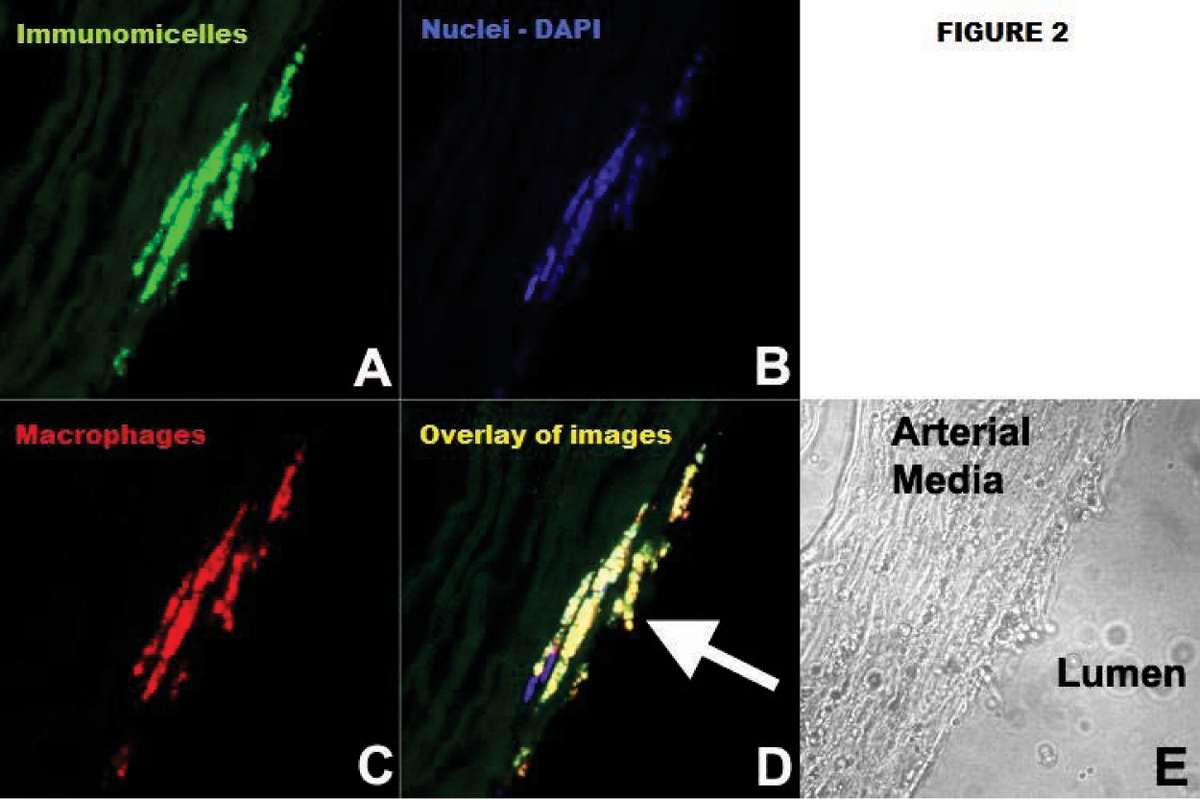
Figure 2

**Figure 3:**
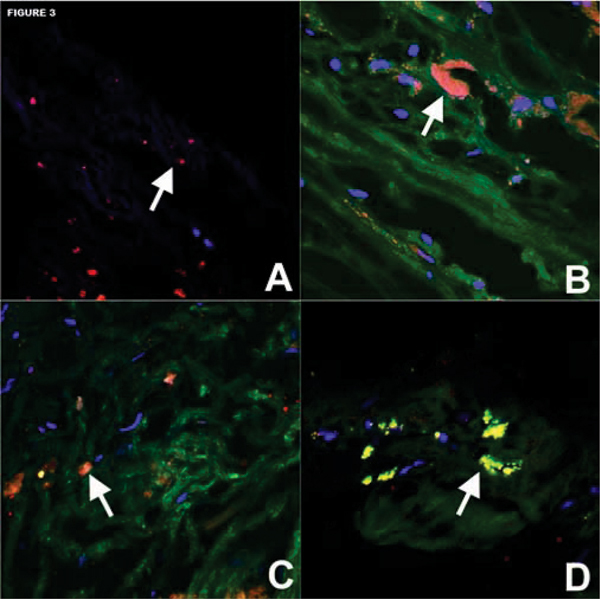
Figure 3

Micelles had a mean diameter of 125 nm, average of 14,900 Gd-ions, and mean relaxivity was 37 mM-1 s-1 at 1.5 T and 37°C. Confocal microscopy and ICP-MS demonstrated significant in vitro uptake of immunomicelles by macrophages while non-targeted micelles had minimal uptake. On T1 imaging, immunomicelles increased CNR by 52.5% (n = 6, p < 0.0001) while Fc-micelles increased CNR by 17.2% (n = 4, p < 0.0001) and micelles increased CNR by 18.7% (n = 6, p = 0.0007). Please see Figure 1. Immunomicelles increased CNR significantly greater than the Fc-micelles or micelles (p = 0.001). Confocal fluorescent microscopy showed that immunomicelles target macrophages in the aortic plaque while the micelles and Fc-micelles are found diffusely throughout the plaque. Please see Figures 2 and 3. Immunomicelles had a greater increase in post-contrast SNR in the fibrous cap compared with the lipid core (p < 0.001) while micelles and Fc-micelles had a greater increase in the lipid core (p < 0.01).

## Conclusion

Macrophage-specific (CD36) immunomicelles bind to human macropages in vitro and improved MR detection and characterization of human aortic atherosclerosis. Thus, immunomicelles could help identify high-risk human plaque.

